# Supplementary material for: Release of Histone H3K4-reading transcription factors from chromosomes in mitosis is independent of adjacent H3 phosphorylation
Source: Nat Commun. 2023 Nov 9;14:7243. doi: 10.1038/s41467-023-43115-3 (PMC10636195; doi:10.1038/s41467-023-43115-3)
Supplement: Supplementary file 1 — Supplementary Information [file 41467_2023_43115_MOESM1_ESM.pdf]

Supplementary Information

Release of Histone H3K4-reading transcription factors from chromosomes in mitosis is independent of adjacent H3 phosphorylation

Rebecca J. Harris, Maninder Heer, Mark D. Levasseur, Tyrell N. Cartwright, Bethany Weston, Jennifer L. Mitchell, Jonathan M. Coxhead, Luke Gaughan, Lisa Prendergast, Daniel Rico, Jonathan M. G. Higgins

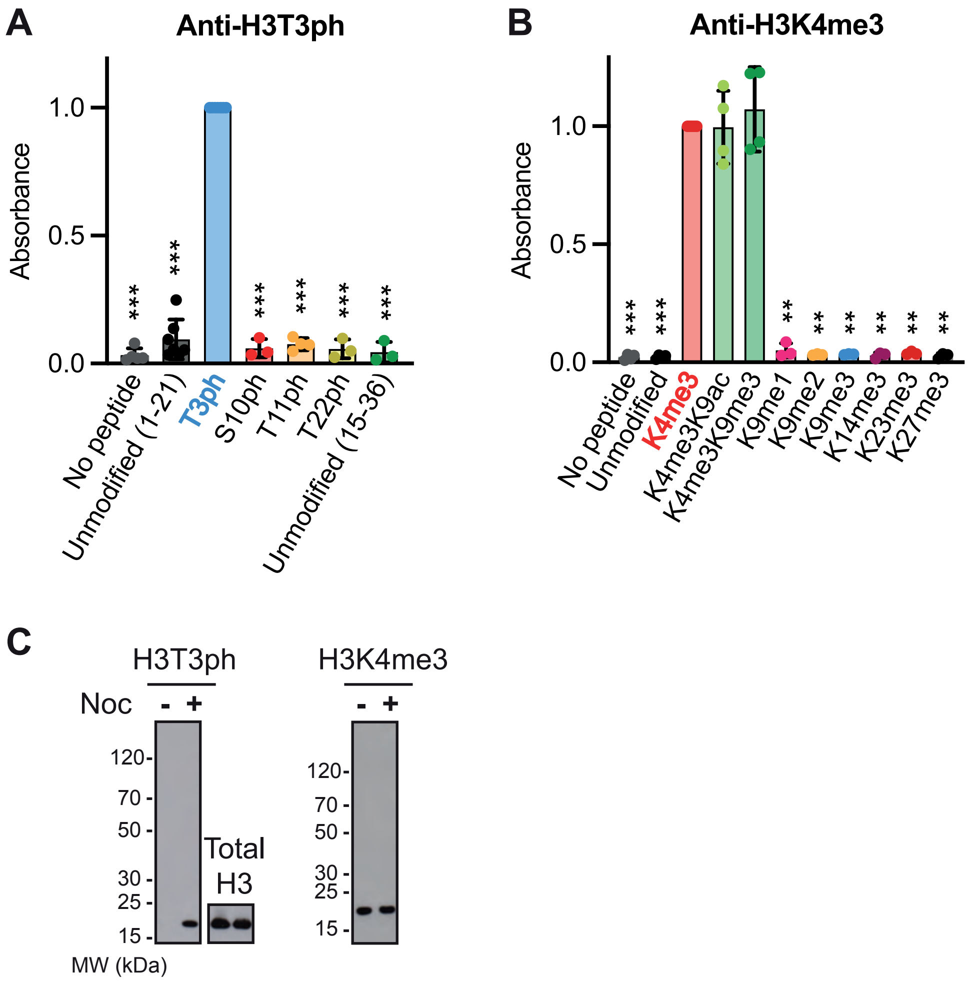

Supplementary Figure 1. Additional antibody characterisation

A. H3T3ph antibody B8634 binding to H3 peptides with various phosphorylations detected by ELISA (for no peptide n = 5; unmodified (1-21) n = 8; T3ph n = 7; S10ph, T22ph, and unmodified (15-36) n = 3; T11ph n = 4).

B. H3K4me3 antibody C42D8 binding to H3 peptides with various methylations detected by ELISA (for no peptide, unmodified, K4me3K9ac, and K4me3K9me3 n = 4; K4me3 n = 6; K9me1, K9me2, K9me3, K14me3, K23me3, and K27me3 n = 3).

Data were normalized to the mean signal of the antibodies on the respective target peptide. Bars represent the mean ± SD. Statistical analysis was carried out using non-normalised data, *** $p < 0.0001$, ** $p < 0.001$, * $p < 0.01$, when compared to binding to the expected modification (H3T3ph or H3K4me3).

C. Immunoblotting of asynchronous and nocodazole-treated (mitotic) HeLa whole cell lysates with H3T3ph antibody B8634 and H3K4me3 antibody C42D8. The total Histone H3 loading control was carried out in parallel with anti-H3T3ph in the same experiment. Similar results were obtained in 3 independent experiments.

Source data including exact p values are provided as a Source Data file.

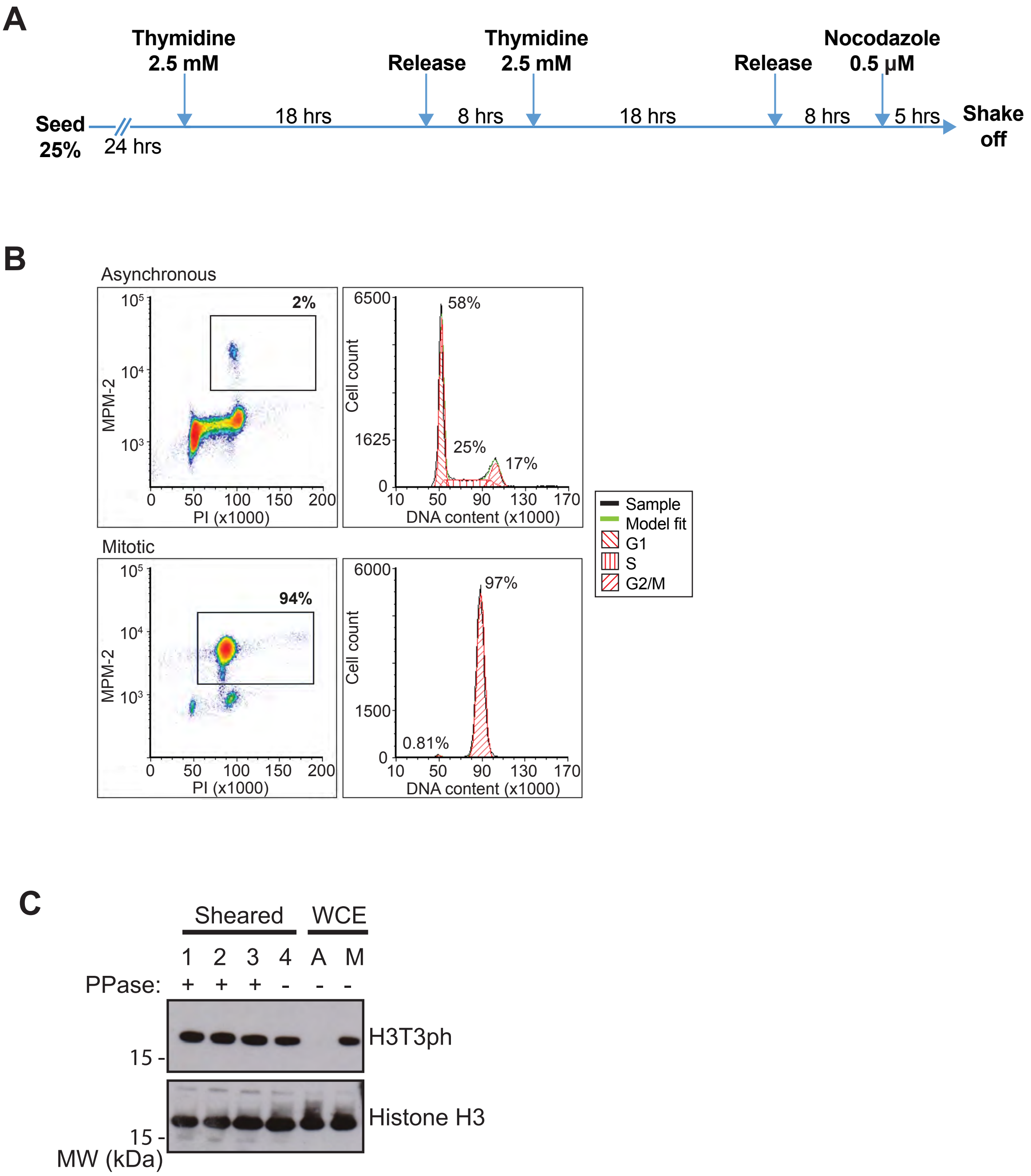

Supplementary Figure 2. Mitotic enrichment of HeLa cells for ChIP-seq

A. Schematic of synchronization method.

B. Representative flow cytometry results of asynchronous cells (top) or cells after synchronization in mitosis (bottom). Percentages indicate the proportion of cells in different cell cycle stages. The mitotic populations, as defined by MPM-2 and propidium iodide (PI) staining, are boxed. Similar results were obtained for the 4 samples used in the ChIP and CIDOP experiments.

C. Immunoblot of sheared chromatin (samples 1 to 4, from n = 4 independent preparations) or whole cell extract (WCE) from asynchronous (A) or synchronized mitotic (M) HeLa cells probed for H3T3ph or total H3. Whether protein phosphatase inhibitors were included during chromatin preparation is indicated. Source data are provided as a Source Data file.

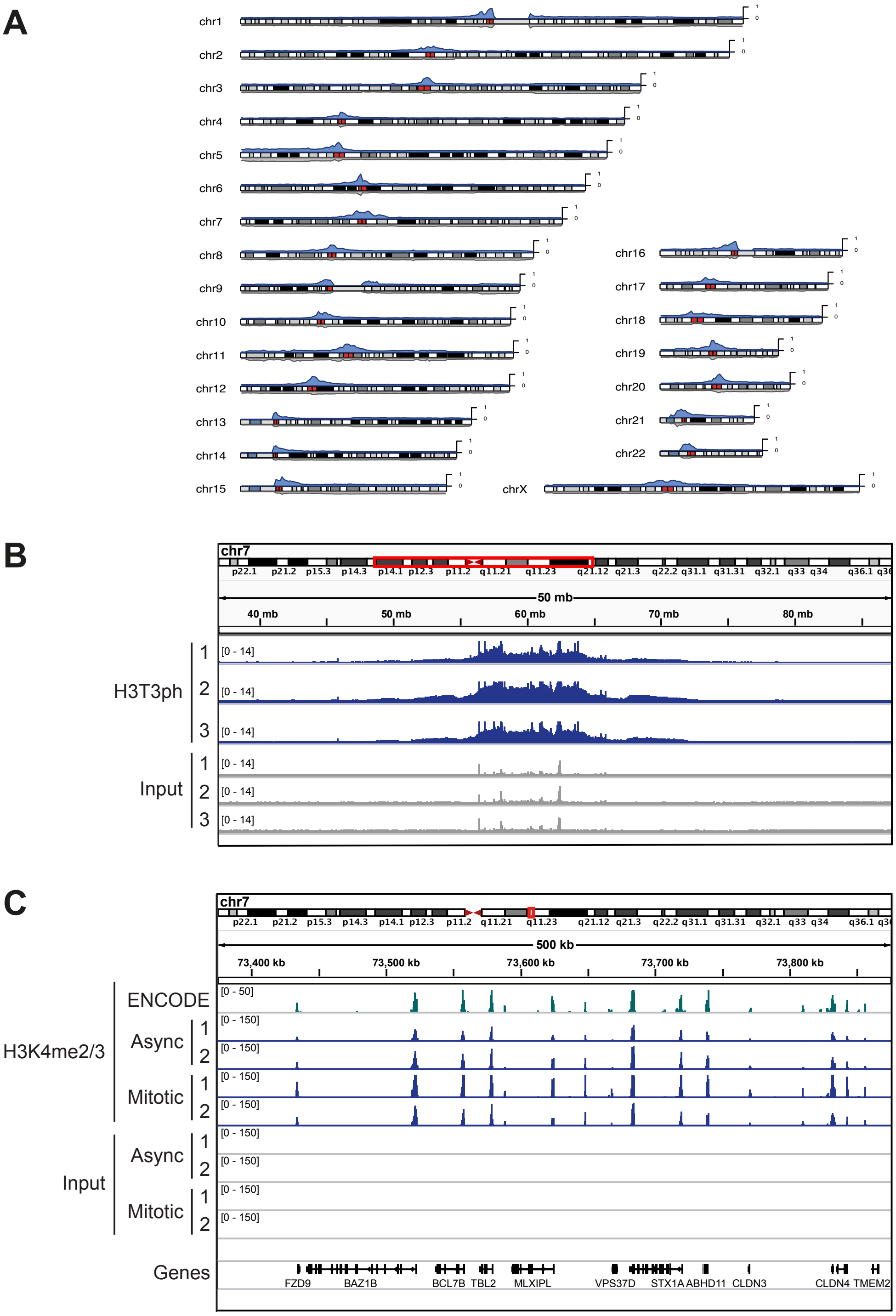

Supplementary Figure 3. ChIP-seq results and comparison with previous ENCODE data

A. Ideograms showing enrichment of H3T3ph at the centromeres of all human chromosomes. Blue tracks represent H3T3ph read data from a single replicate aligned to GRCh38.p12. Corresponding input reads are plotted in gray. Gaps at the centromeres of chromosomes 1, 9, and 16 reflect incomplete sequence information for these regions in GRCh38.p12.

B. Integrative Genomics Viewer (IGV) tracks of H3T3ph ChIP-seq for a 50 MB region of chromosome 7 encompassing the centromere (GRCh38.p12). Read coverages for biological replicates 1 to 3 are shown (H3T3ph in blue, inputs in grey). Reads mapping to multiple sites were randomly assigned.

C. IGV ChIP-seq tracks for a 500 kb region of chromosome 7. H3K4me3 ChIP-seq from the ENCODE project (ENCFF489CIY; fold change over control; green), H3K4me2/3 ChIP-seq biological replicates 1 and 2 generated in the present study (blue), and the corresponding inputs (grey), for both the asynchronous and mitotic HeLa cells are shown by read coverage. The bottom row shows genes present in this region.

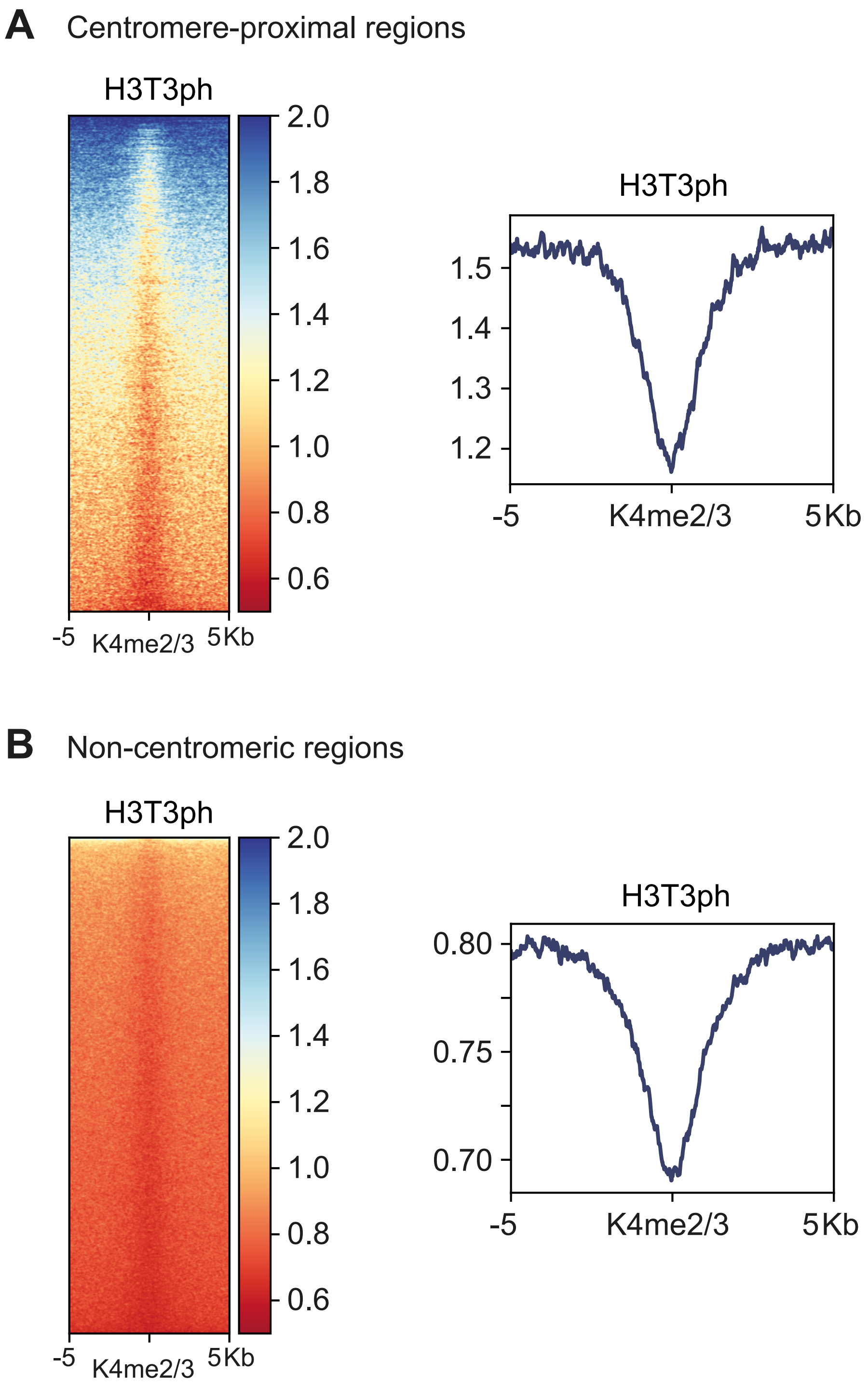

Supplementary Figure 4. ChIP-seq for H3T3ph in HeLa cells

A. Heatmap (left) and metagene plot (right) of H3T3ph ChIP-seq at 10 kb regions centered on mitotic H3K4me2/3 ChIP-seq peaks at centromere-proximal regions.

B. As for A, but showing non-centromeric regions. Vertical scales show enrichment scores.

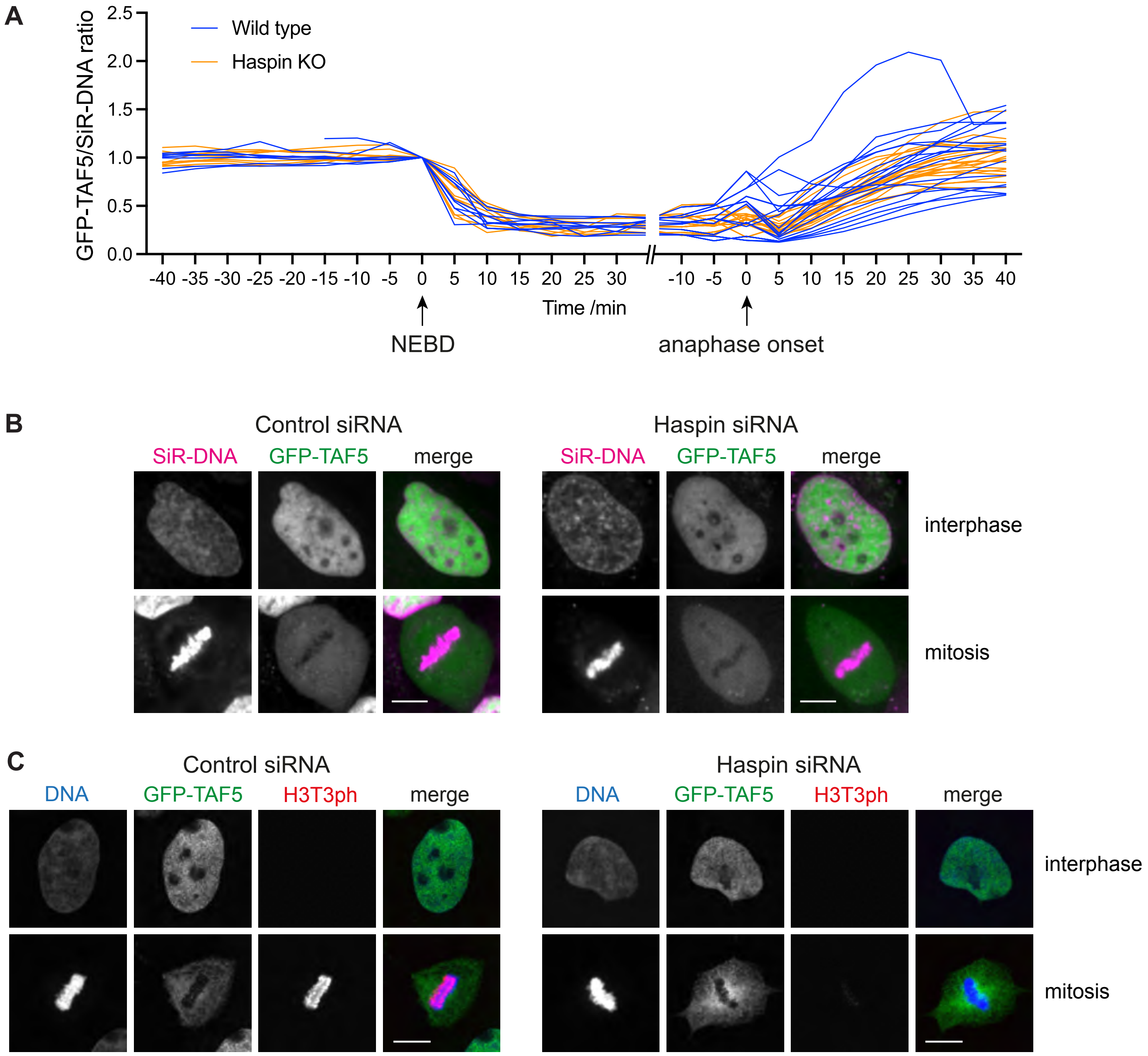

Supplementary Figure 5. Haspin loss or inhibition does not influence the displacement of GFP-TAF5 from chromosomes in mitosis

A. Quantification of GFP-TAF5/SiR-DNA ratio during live imaging of GFP-TAF5-expressing wild type and Haspin knockout HeLa cells. DNA was stained with SiR-DNA, images were taken every 5 min, and times are stated in minutes before and after nuclear envelope breakdown (NEBD) and anaphase onset as appropriate. Traces for individual wild type (n = 9) and Haspin knockout (n = 10) HeLa cells imaged in 3 independent experiments are shown. Source data are provided as a Source Data file.

B. Live imaging of U2OS cells expressing GFP-TAF5 (green) after control or Haspin RNAi. DNA was stained with SiR-DNA (magenta). For display, intensities of GFP and SiR-DNA images were adjusted separately for interphase and mitosis, but equally for control and Haspin RNAi. Representative cells from 3 independent transfections are shown. All 10 Haspin siRNA-treated cells imaged showed clear exclusion of GFP-TAF5 from mitotic chromosomes, similar to that in 16 imaged control cells.

C. Immunofluorescence microscopy (with formaldehyde fixation) for DNA (blue), GFP-TAF5 (green), and H3T3ph (red) in U2OS cells after control or Haspin RNAi. This experiment was carried out in parallel with the live imaging in (**B**), and showed that all 19 mitotic cells examined that had been treated with Haspin siRNA showed exclusion of GFP-TAF5 from chromosomes, and had H3T3ph levels at least 90% lower than the mean of those in control siRNA-treated cells (mean remaining H3T3ph = 4%). A second independent experiment gave similar results, and the results were confirmed using Haspin inhibitor treatment (Supplementary Figure 6A), and in live and fixed HeLa Haspin KO cells (Figure 4). Scale bars = 10 μm.

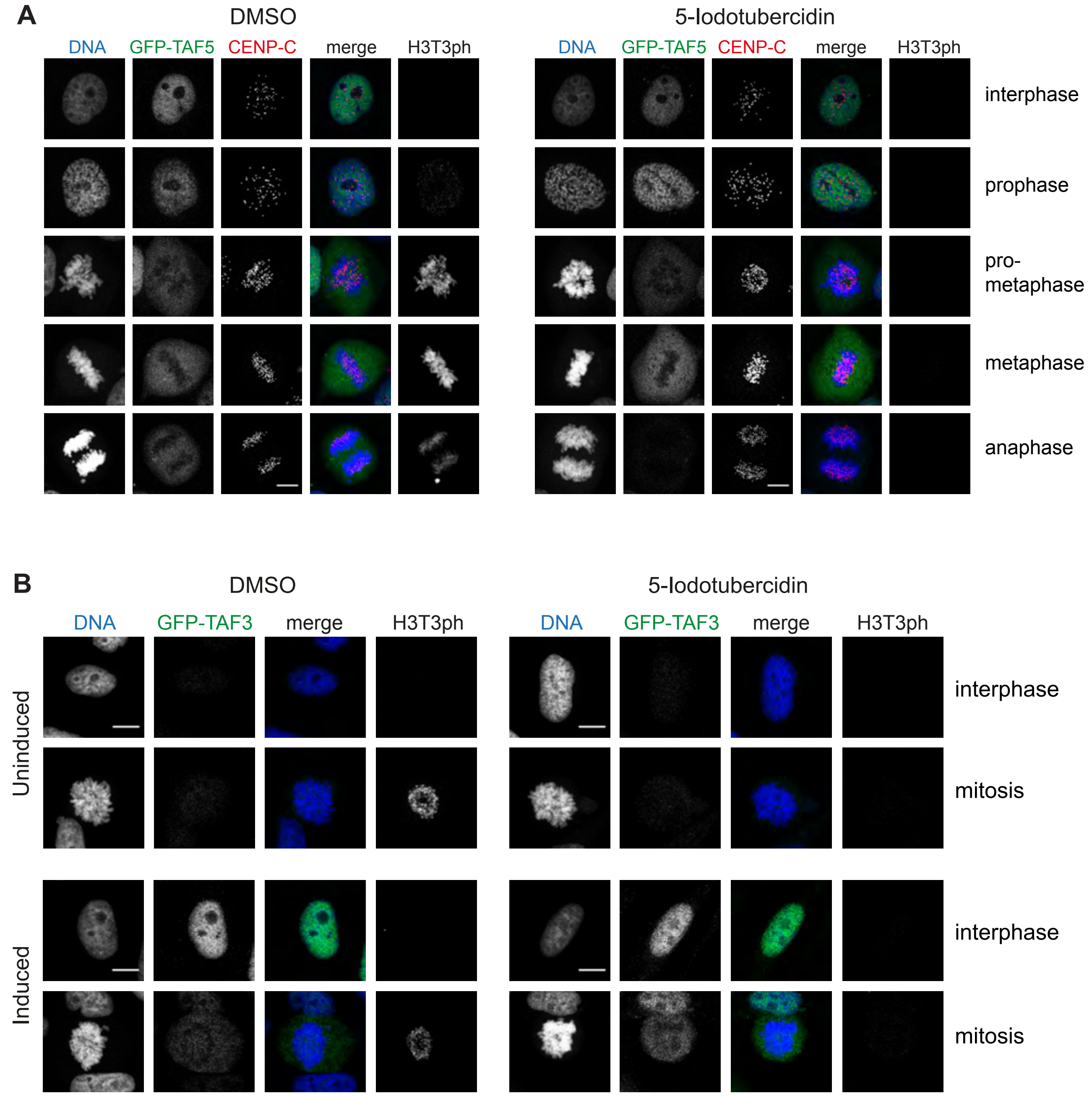

Supplemental Figure 6. Haspin inhibition does not influence the displacement of GFP-TAF5 or GFP-TAF3 from chromosomes in mitosis

A. Immunofluorescence microscopy (with formaldehyde fixation) for DNA (blue), GFP (green), CENP-C (centromeres, red), and H3T3ph (gray) in GFP-TAF5 expressing U2OS cells that were treated, or not treated, with 1 μM Haspin inhibitor 5-iodotubercidin. This experiment was performed once, but was confirmed with formaldyde fixation, and using live imaging of Haspin KO cells (see Figure 4).

B. Immunofluorescence microscopy (with formaldehyde fixation) for DNA (blue), GFP (green), and H3T3ph (gray) in HeLa cells inducibly expressing GFP-TAF3 that were treated, or not treated, with 1 μM Haspin inhibitor 5-iodotubercidin. GFP-TAF3 expression was induced with doxycycline where indicated. Similar results were obtained in a second independent experiment, and the results were confirmed for endogenous TAF3 (see Figure 5). Scale bars = 10 μm.

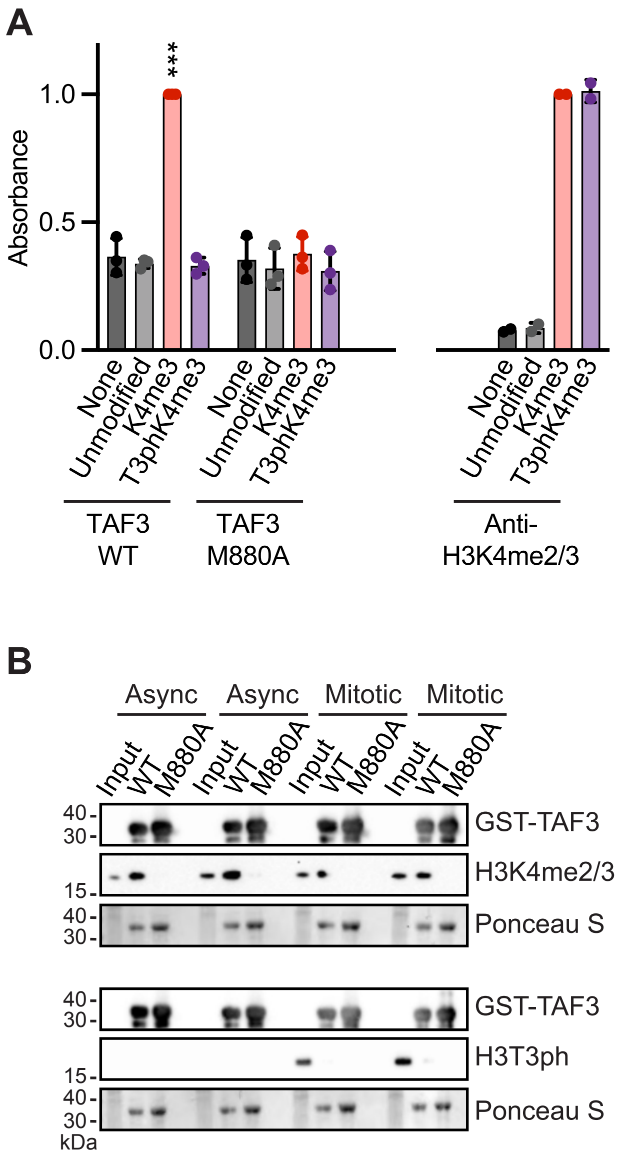

Supplementary Figure 7. The influence of H3T3ph on GST-TAF3 PHD finger binding to H3K4me3

A. Left: wild type (WT) and M880A mutant GST-TAF3 PHD finger binding to H3 peptides with various modifications (n = 3). Right: Controls showing H3K4me2/3 antibody C42D8 binding to the same H3 peptides (n = 2). Data were normalized to the mean signal of GST-TAF3 or H3K4me2/3 antibody binding to H3K4me3 peptide. Bars represent mean ± SD. Statistical analysis was carried out where $n > 2$, using non-normalised data and a repeated measures one-way ANOVA with Dunnett's adjustment for multiple comparisons. *** $p < 0.0001$, ** $p < 0.001$, * $p < 0.01$, when compared to binding in the absence of peptide.

B. Wild type, but not M880A mutant, GST-TAF3 PHD finger immunoprecipitates Histone H3 carrying H3K4me2/3, but not H3T3ph, from asynchronous and mitotic HeLa cell extracts. This experiment was perfomed once with 2 independent chromatin samples for both async and mitosis. Source data including exact p values are provided as a Source Data file.

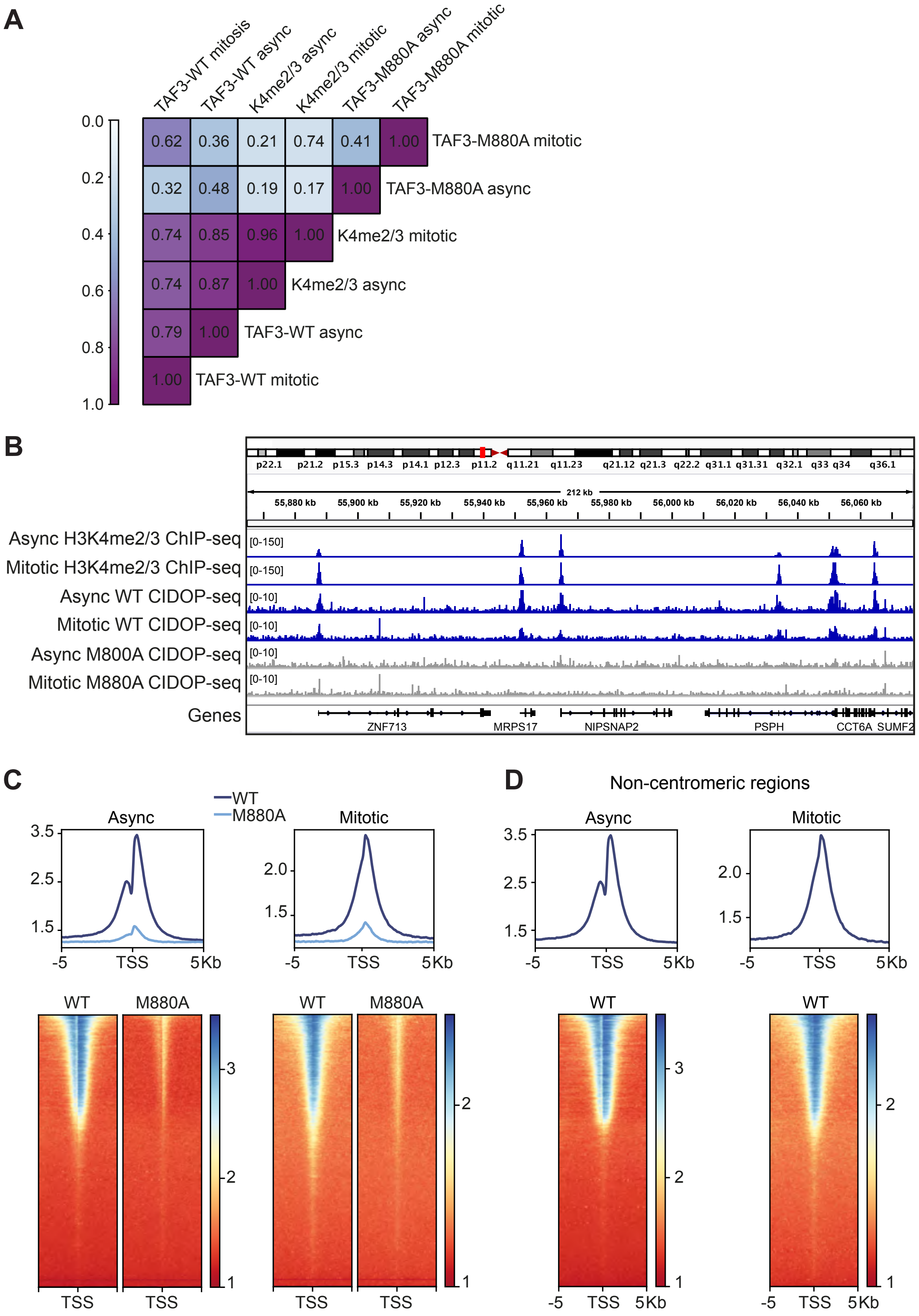

Supplementary Figure 8. CIDOP-seq of GST-TAF3 PHD finger binding to chromatin from asynchronous and mitotic HeLa cells

A. Pearson correlation coefficients between genome wide read coverage of TAF3 PHD CIDOP-seq (WT or M880A mutant) and H3K4me2/3 ChIP-seq for both asynchronous and mitotic-enriched HeLa cells.

B. Representative IGV tracks of a 212 kb region of chromosome 7, showing TAF3 PHD (WT or M880A mutant) CIDOP-seq and H3K4me2/3 ChIP-seq for asynchronous and mitotic-enriched HeLa cells.

C. WT and M880A mutant TAF3 PHD CIDOP-seq enrichment at TSSs genome wide. Results from both asynchronous and mitotic-enriched HeLa cells are shown metagene plots (top) and as heatmaps (bottom). Regions of 10 kb centered at TSSs are shown.

D. TAF3 PHD enrichment across TSSs that are not centromere-proximal (i.e. those on chromosome arms where H3T3ph is low). Metagene plots (top) and heatmaps (bottom) show TAF3 PHD binding at TSSs for both asynchronous and mitotic-enriched CIDOP-seq HeLa cells. Vertical scales show enrichment scores.

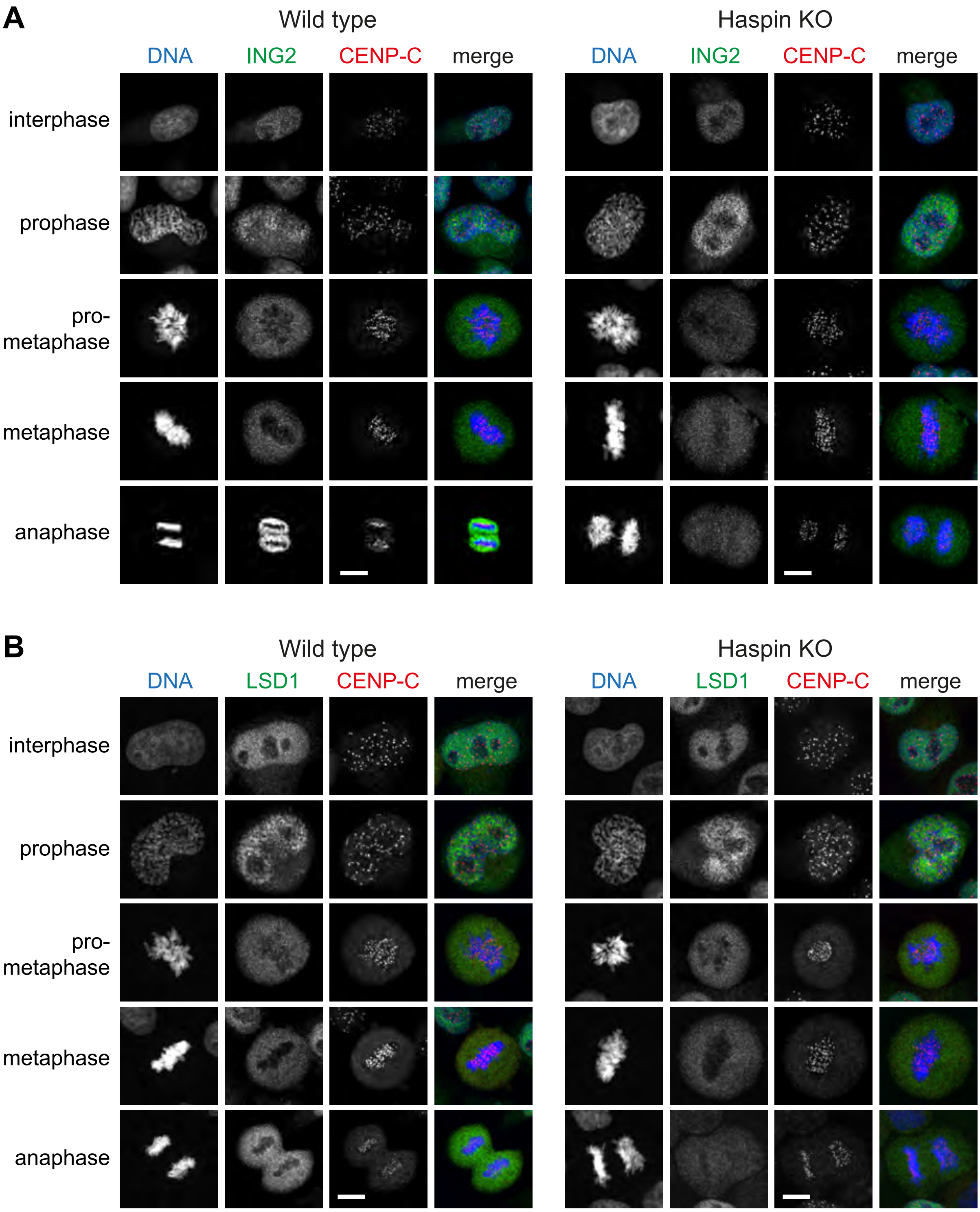

Supplementary Figure 9. Haspin knockout does not influence the displacement of endogenous ING2 or LSD1 from chromosomes in mitosis

A. Immunofluorescence microscopy (with formaldehyde fixation) for DNA (blue), ING2 (green), and CENP-C (centromeres, red) in wild type and Haspin knockout HeLa cells.

B. As for A, but staining for LSD1 (green).

Both experiments were carried out independently 3 times each. Scale bars = 10 µm.

Supplementary Table 1
Mammalian H3K4-reading proteins that are displaced by H3T3ph *in vitro*.

Reader protein	Histone mark	Supplementary references
**AIRE** PHD1 domain	H3K4me0/1	1,2
**BHC80** (**PHF21A**) PHD domain	H3K4me0	2,3
**BHC80L** (**PHF21B**) PHD domain	H3K4me1	4
**BPTF** PHD domain	H3K4me3	3,5
**BRPF2** (**BRD1**) PHD1 domain	H3K4me0	6
**CFP1** PHD domain (**Spp1** in *S. pombe*)	H3K4me2/3	7
**CHD1** DCD domain	H3K4me3	5,8,9
**CHD4** PHD domain	H3K4me0	10,11
**CHD5** PHD domain	H3K4me0	11,12
**DIDO** PHD domain (**Bye1** in *S. cerevisiae*)	H3K4me3	9,11,13-15
**DNMT3A** ADD domain	H3K4me0/1	16,17
**DNMT3B** ADD domain	H3K4me0/1	16,17
**DNMT3L** ADD domain	H3K4me0	17
**DPF2** PHD domain	H3K4me0	11
**ING1** PHD domain	H3K4me3	9
**ING2** PHD domain	H3K4me3	2,3
**ING4** PHD domain	H3K4me3	3
**KDM4A** DTD domain	H3K4me3	9,18
**KDM5B** PHD1 domain	H3K4me0	19
**KDM5B** PHD3 domain	H3K4me3	19
**KDM7A** PHD domain	H3K4me3	11
**MLL1** SET domain	H3K4me0	20
**MLL5** PHD domain	H3K4me3	11,21
**ORC1b** PHD domain (*Arabidopsis*)	H3K4me0	22
**PHF8** PHD domain	H3K4me3	9
**PHRF1** PHD domain	H3K4me0	11
**RAG2** PHD domain	H3K4me3	2,5,9
**SGF29** tandem Tudor domain	H3K4me3	23
**SP140** PHD domain	H3K4me0	24
**TAF3** PHD domain	H3K4me2/3	3,9,23,25
**TRIM66** PHD domain	H3K4me0	11
**WDR5** WD40 repeats	H3K4me0/1/2/3	26,27

Supplementary References

1. Chignola, F. *et al.* The solution structure of the first PHD finger of autoimmune regulator in complex with non-modified histone H3 tail reveals the antagonistic role of H3R2 methylation. *Nucleic Acids Res* **37**, 2951-2961 (2009).
2. Garske, A. L. *et al.* Combinatorial profiling of chromatin binding modules reveals multisite discrimination. *Nat Chem Biol* **6**, 283-290 (2010).
3. Varier, R. A. *et al.* A phospho/methyl switch at histone H3 regulates TFIID association with mitotic chromosomes. *EMBO J* **29**, 3967-3978 (2010).
4. Basu, A. *et al.* Phf21b imprints the spatiotemporal epigenetic switch essential for neural stem cell differentiation. *Genes Dev* **34**, 1190-1209 (2020).
5. Fuchs, S. M., Krajewski, K., Baker, R. W., Miller, V. L. & Strahl, B. D. Influence of combinatorial histone modifications on antibody and effector protein recognition. *Curr Biol* **21**, 53-58 (2011).
6. Qin, S. *et al.* Recognition of unmodified histone H3 by the first PHD finger of bromodomain-PHD finger protein 2 provides insights into the regulation of histone acetyltransferases monocytic leukemic zinc-finger protein (MOZ) and MOZ-related factor (MORF). *J Biol Chem* **286**, 36944-36955 (2011).
7. He, C. *et al.* Structural basis for histone H3K4me3 recognition by the N-terminal domain of the PHD finger protein Spp1. *Biochem J* **476**, 1957-1973 (2019).
8. Flanagan, J. F. *et al.* Double chromodomains cooperate to recognize the methylated histone H3 tail. *Nature* **438**, 1181-1185 (2005).
9. Gatchalian, J. *et al.* Chromatin condensation and recruitment of PHD finger proteins to histone H3K4me3 are mutually exclusive. *Nucleic Acids Res* **44**, 6102-6112 (2016).
10. Mansfield, R. E. *et al.* Plant homeodomain (PHD) fingers of CHD4 are histone H3-binding modules with preference for unmodified H3K4 and methylated H3K9. *J Biol Chem* **286**, 11779-11791 (2011).
11. Jain, K. *et al.* Characterization of the plant homeodomain (PHD) reader family for their histone tail interactions. *Epigenet Chromatin* **13**, 3 (2020).
12. Oliver, S. S. *et al.* Multivalent recognition of histone tails by the PHD fingers of CHD5. *Biochemistry* **51**, 6534-6544 (2012).
13. Gatchalian, J. *et al.* Dido3 PHD modulates cell differentiation and division. *Cell Rep* **4**, 148-158 (2013).
14. Kinkelin, K. *et al.* Structures of RNA polymerase II complexes with Bye1, a chromatin-binding PHF3/DIDO homologue. *Proc Natl Acad Sci U S A* **110**, 15277-15282 (2013).
15. Tencer, A. H. *et al.* A Unique pH-Dependent Recognition of Methylated Histone H3K4 by PPS and DIDO. *Structure* **25**, 1530-1539 e1533 (2017).
16. Zhang, Y. *et al.* Chromatin methylation activity of Dnmt3a and Dnmt3a/3L is guided by interaction of the ADD domain with the histone H3 tail. *Nucleic Acids Res* **38**, 4246-4253 (2010).
17. Noh, K. M. *et al.* Engineering of a Histone-Recognition Domain in Dnmt3a Alters the Epigenetic Landscape and Phenotypic Features of Mouse ESCs. *Mol Cell* **59**, 89-103 (2015).
18. Su, Z. *et al.* Reader domain specificity and lysine demethylase-4 family function. *Nat Commun* **7**, 13387 (2016).
19. Klein, B. J. *et al.* The histone-H3K4-specific demethylase KDM5B binds to its substrate and product through distinct PHD fingers. *Cell Rep* **6**, 325-335 (2014).
20. Southall, S. M., Wong, P. S., Odho, Z., Roe, S. M. & Wilson, J. R. Structural basis for the requirement of additional factors for MLL1 SET domain activity and recognition of epigenetic marks. *Mol Cell* **33**, 181-191 (2009).
21. Ali, M. *et al.* Molecular basis for chromatin binding and regulation of MLL5. *Proc Natl Acad Sci U S A* **110**, 11296-11301 (2013).
22. Li, S. *et al.* Structural Basis for the Unique Multivalent Readout of Unmodified H3 Tail by Arabidopsis ORC1b BAH-PHD Cassette. *Structure* **24**, 486-494 (2016).
23. Shanle, E. K. *et al.* Histone peptide microarray screen of chromo and Tudor domains defines new histone lysine methylation interactions. *Epigenet Chromatin* **10**, 12 (2017).
24. Zhang, X., Zhao, D., Xiong, X., He, Z. & Li, H. Multifaceted Histone H3 Methylation and Phosphorylation Readout by the Plant Homeodomain Finger of Human Nuclear Antigen Sp100C. *J Biol Chem* **291**, 12786-12798 (2016).
25. Kungulovski, G., Mauser, R., Reinhardt, R. & Jeltsch, A. Application of recombinant TAF3 PHD domain instead of anti-H3K4me3 antibody. *Epigenet Chromatin* **9**, 11 (2016).
26. Couture, J. F., Collazo, E. & Trievel, R. C. Molecular recognition of histone H3 by the WD40 protein WDR5. *Nat Struct Mol Biol* **13**, 698-703 (2006).
27. Klingberg, R. *et al.* Analysis of phosphorylation-dependent protein-protein interactions of histone H3. *ACS Chem Biol* **10**, 138-145 (2015).
