# Supplementary material for: Release of Histone H3K4-reading transcription factors from chromosomes in mitosis is independent of adjacent H3 phosphorylation
Source: Nat Commun. 2023 Nov 9;14:7243. doi: 10.1038/s41467-023-43115-3 (PMC10636195; doi:10.1038/s41467-023-43115-3)
Supplement: Supplementary file 3 — Description of Additional Supplementary Files [file 41467_2023_43115_MOESM3_ESM.pdf]

Description of Additional Supplementary Files

File Name: Supplementary Movie 1, related to Figure 4.
Description: Live imaging of a GFP-TAF5-expressing wild type HeLa cell progressing through mitosis.

File Name: Supplementary Movie 2, related to Figure 4.
Description: Live imaging of a GFP-TAF5-expressing Haspin-knockout HeLa cell progressing through mitosis. For both movies: Left panel: grayscale image of GFP fluorescence. Middle panel: grayscale image of DNA stained with SiR-DNA. Right panel: merged images of GFP (green) and DNA (magenta). Images were taken every 5 min.
