# Supplementary material for: Release of Histone H3K4-reading transcription factors from chromosomes in mitosis is independent of adjacent H3 phosphorylation
Source: Nat Commun. 2023 Nov 9;14:7243. doi: 10.1038/s41467-023-43115-3 (PMC10636195; doi:10.1038/s41467-023-43115-3)
Supplement: Supplementary file 6 — Reporting Summary [file 41467_2023_43115_MOESM6_ESM.pdf]

Corresponding author(s): Jonathan Higgins; Daniel Rico

Last updated by author(s): Oct 18, 2023

Reporting Summary

Nature Portfolio wishes to improve the reproducibility of the work that we publish. This form provides structure for consistency and transparency in reporting. For further information on Nature Portfolio policies, see our Editorial Policies and the Editorial Policy Checklist.

Statistics

For all statistical analyses, confirm that the following items are present in the figure legend, table legend, main text, or Methods section.

n/a	Confirmed	
☐	☒	The exact sample size (*n*) for each experimental group/condition, given as a discrete number and unit of measurement
☐	☒	A statement on whether measurements were taken from distinct samples or whether the same sample was measured repeatedly
☐	☒	The statistical test(s) used AND whether they are one- or two-sided<br>*Only common tests should be described solely by name; describe more complex techniques in the Methods section.*
☒	☐	A description of all covariates tested
☐	☒	A description of any assumptions or corrections, such as tests of normality and adjustment for multiple comparisons
☐	☒	A full description of the statistical parameters including central tendency (e.g. means) or other basic estimates (e.g. regression coefficient) AND variation (e.g. standard deviation) or associated estimates of uncertainty (e.g. confidence intervals)
☐	☒	For null hypothesis testing, the test statistic (e.g. *F*, *t*, *r*) with confidence intervals, effect sizes, degrees of freedom and *P* value noted<br>*Give P values as exact values whenever suitable.*
☒	☐	For Bayesian analysis, information on the choice of priors and Markov chain Monte Carlo settings
☒	☐	For hierarchical and complex designs, identification of the appropriate level for tests and full reporting of outcomes
☐	☒	Estimates of effect sizes (e.g. Cohen's *d*, Pearson's *r*), indicating how they were calculated

Our web collection on statistics for biologists contains articles on many of the points above.

Software and code

Policy information about availability of computer code

Data collection	For fluorescence microscopy we used LasX v3 software (Leica) or Elements 5.21.03 or Elements 5.22 software (Nikon).
Data analysis	For analyzing ELISA and kinase assay data we used Microsoft Excel 16.26, and statistical analyses were carried out in Prism 9.4.1 (GraphPad) or Microsoft Excel 16.26. For flow cytometry data, we used FCS Express 7 (De Novo Software). For image quantification, we used ImageJ 2.3.0. For sequencing data analysis, we used FastQC v0.11.7, MultiQC v1.7216, Bowtie2 v2.3.4.2, Samtools v1.9, bedtools v2.29.2, Sambamba v0.7.1, deepTools2 v3.5.1, MACS2 v2.1.1.20160309, bedops v2.4.39, KaryoploteR v1.22.0, R v4.2.1 or v4.2.2, IGV v2.4.16, ChromHMM v1.20, and WebGestalt 2019, as detailed in the Methods.

For manuscripts utilizing custom algorithms or software that are central to the research but not yet described in published literature, software must be made available to editors and reviewers. We strongly encourage code deposition in a community repository (e.g. GitHub). See the Nature Portfolio guidelines for submitting code & software for further information.

Data

Policy information about availability of data

All manuscripts must include a data availability statement. This statement should provide the following information, where applicable:

- Accession codes, unique identifiers, or web links for publicly available datasets
- A description of any restrictions on data availability
- For clinical datasets or third party data, please ensure that the statement adheres to our policy

Raw and processed ChIP-seq and CIDOP-seq data generated in this study have been deposited in the public Gene Expression Omnibus under GEO accession code GSE226768 [https://www.ncbi.nlm.nih.gov/geo/query/acc.cgi?&acc=GSE226768]. The human reference genome GRCh38.p12 (GCA_000001405.27) used in this study is available from the National Center for Biotechnology Information [https://www.ncbi.nlm.nih.gov/datasets/genome/GCF_000001405.27/], and the HeLa H3K4me3 data used in Supplementary Figure 3 is available from the ENCODE Portal [https://www.encodeproject.org/files/ENCFF489CIY/]. Quantitative data from ELISA, kinase and binding assays, live cell imaging, and immunofluorescence microscopy are provided in the Source Data file.

Research involving human participants, their data, or biological material

Policy information about studies with human participants or human data. See also policy information about sex, gender (identity/presentation), and sexual orientation and race, ethnicity and racism.

Reporting on sex and gender	N/A
Reporting on race, ethnicity, or other socially relevant groupings	N/A
Population characteristics	N/A
Recruitment	N/A
Ethics oversight	N/A

Note that full information on the approval of the study protocol must also be provided in the manuscript.

Field-specific reporting

Please select the one below that is the best fit for your research. If you are not sure, read the appropriate sections before making your selection.

☒ Life sciences ☐ Behavioural & social sciences ☐ Ecological, evolutionary & environmental sciences

For a reference copy of the document with all sections, see nature.com/documents/nr-reporting-summary-flat.pdf

Life sciences study design

All studies must disclose on these points even when the disclosure is negative.

Sample size	Sample sizes were set at a minimum of 3, except for ChIP-seq and CIDOP-seq (where n=2 when experiment was essentially confirming prior results, and otherwise n=3). For other experiments, sample sizes were for ELISAs (n=3 to 8), kinase assays (n=3 to 4), cell imaging (9 to 20 cells). Sample sizes were not based on power calculations, but were predetermined in line with standards in the field, together with the practicality of handling, cost of reagents etc.
Data exclusions	When quantifying live cell imaging experiments, cells with low intensity tagged protein fluorescence were excluded (integrated pixel intensities in red or green channel of below 200,000 per cell).
Replication	ChIP-seq and CIDOP-seq experiments were carried out 2 or 3 times in independent experiments. The results of all such experiments are reported. For TFIID localisation studies, we confirmed the results in similar experiments in multiple ways: by visualising GFP-TAF5 in living HeLa Haspin KO cells (n = 3) or in U2OS cells upon Haspin RNAi (n = 3); by immunofluorescence staining of GFP-TAF5 in HeLa Haspin KO cells (n = 1) and in U2OS cells upon Haspin RNAi (n = 2) or inhibitor treatment (n = 1); by immunofluorescence staining of GFP-TAF3 in HeLa cells treated with Haspin inhibitor (n = 2); and by immunofluorescence staining of endogenous TAF3 in Haspin KO cells (n = 1). For ING2, DIDO and LSD1 immunofluorescence experiments, the results of staining were confirmed in two fixation conditions, PFA (n = 3) and methanol (n = 1 or 2, as stated in figure legends), with the exception of DIDO where staining following formaldehyde fixation was ineffective. Immunoblotting results were confirmed with n = 2 to 4 separate protein preparations, as stated in figure legends.
Randomization	Randomization was not relevant because experiments involved treatments of cells derived from a single cell culture.
Blinding	Samples were not strictly blinded, but quantitative analysis was largely done using automated systems, and thus was effectively blinded.

Reporting for specific materials, systems and methods

We require information from authors about some types of materials, experimental systems and methods used in many studies. Here, indicate whether each material, system or method listed is relevant to your study. If you are not sure if a list item applies to your research, read the appropriate section before selecting a response.

Materials & experimental systems

n/a	Involved in the study
☐	☒ Antibodies
☐	☒ Eukaryotic cell lines
☒	☐ Palaeontology and archaeology
☒	☐ Animals and other organisms
☒	☐ Clinical data
☒	☐ Dual use research of concern
☒	☐ Plants

Methods

n/a	Involved in the study
☐	☒ ChIP-seq
☒	☐ Flow cytometry
☒	☐ MRI-based neuroimaging

Antibodies

Antibodies used

Rabbit monoclonal antibodies:
H3K4me3 (C42D8; Cell Signaling Technology #9751, Lot 10)

Rabbit polyclonal antibodies:
H3T3ph (B8634; custom antibody generated by Biosource, see ref 35)
Histone H3 (Abcam ab1791, Lot GR300976-2)
Dido/DATF1 (Invitrogen PA5-101330, Lot XB3517451A)
ING2 (Sigma HPA019486, Lot R08441)
LSD1/KDM1A (Abcam ab17721, Lot GR278845-1)
GFP (Invitrogen A-11122, Lot 1293114)

Mouse monoclonal antibodies:
Histone H3T3ph (16B2; gift from Hiroshi Kimura, see ref 37)
TAF3 (39TA-2F5; Invitrogen MA3-074, Lot SC242976)
GST (GST 3-4C; Invitrogen 13-6700, Lot VE304933)
Cy5-conjugated MPM2 (Sigma 16-220, Lot 2762297)

Chicken polyclonal antibodies:
GFP (Abcam ab13970, Lot GR3190550-8)

Guinea pig polyclonal antibodies:
CENP-C (MBL Life Science PD030, Lot 007)

Donkey polyclonal antibodies:
anti-mouse Alexa Fluor 488 (Invitrogen A-21202, Lot 1423052)
anti-mouse Alexa Fluor Plus 594 (Invitrogen A-32744, Lot UL294787)
anti-rabbit Alexa Fluor Plus 594 (Invitrogen A-32754, Lot UI290268)

Goat polyclonal antibodies:
anti-rabbit-IgG Alexa Fluor Plus 488 (Invitrogen A-32790, Lot UL293143)
anti-guinea pig IgG Alexa Fluor 647 (Invitrogen A-21450, Lot 1711474)
anti-chicken IgG Alexa Fluor 488 (Invitrogen A-11039, Lot 1869581)
anti-rabbit IgG-HRP (Cell Signaling Technology #7074, Lot 28)

Horse polyclonal antibodies:
anti-mouse IgG-HRP (Cell Signaling Technology #7076, Lot 33)

Validation

H3T3ph (B8634) and H3K4me2/3 (C42D8) antibodies are validated in the present study by peptide ELISAs and immunoblotting, and by prior studies (ref 35, 59-62). H3T3ph (16B2) antibody was validated by peptide ELISA and by Haspin-dependence in immunoblotting (ref 37). Histone H3 antibody (Abcam ab1791) has been validated, including by peptide competition (see Abcam website); Dido/DATF1 (Invitrogen PA5-101330) recognises a protein of the expected size and location by immunoblotting and immunofluorescence (see Invitrogen website); ING2 (Sigma HPA019486) is validated by the Human Protein Atlas (https://www.proteinatlas.org/ENSG00000168556-ING2/summary/antibody); LSD1/KDM1A (Abcam ab17721) by genetic knockout and immunoblotting (see Abcam website); and TAF3 (39TA-2F5; Invitrogen MA3-074) has been validated by transfection and immunoprecipitation (PMID: 18549481). CENP-C antibody (MBL PD030) recognises a protein of the expected size and location by immunoblotting, immunoprecipitation, and immunofluorescence (see MBL website). MPM2 (Sigma 16-220) is mitosis-specific by immunofluorescence and immunoblotting (see Sigma website).

Eukaryotic cell lines

Policy information about cell lines and Sex and Gender in Research

Cell line source(s)	HeLa S3 cells were obtained from the ATCC (ATCC CCL 2.2); parental and Haspin knockout D2 Hela cells from Dr Fangwei Wang (Zhejiang University, China; ref 68); human GFP-TAF5 U2OS cells, doxycycline-inducible mouse GFP-TAF3 HeLa FRT and human GFP-TAF5 HeLa FRT cells from Dr Marc Timmers (German Cancer Research Center, University of Freiburg; ref 47, 49, 102).
Authentication	HeLa S3 were authenticated using STR profiling by the ATCC and not subsequently retested for authenticity. Other cell lines were not tested.
Mycoplasma contamination	Cells were free from mycoplasma as determined by fluorescence microscopy with DNA dyes and periodic PCR-based assay.
Commonly misidentified lines (See ICLAC register)	Commonly misidentified cell lines were not used in this study.

Plants

Seed stocks	N/A
Novel plant genotypes	N/A
Authentication	N/A

ChIP-seq

Data deposition

☒ Confirm that both raw and final processed data have been deposited in a public database such as GEO.

☒ Confirm that you have deposited or provided access to graph files (e.g. BED files) for the called peaks.

Data access links
May remain private before publication.

https://www.ncbi.nlm.nih.gov/geo/query/acc.cgi?acc=GSE226768

Files in database submission

Accession Title Release date Status Supplementary files

GSE226768 Release of Histone H3K4-reading transcription factors from chromosomes in mitosis is independent of adjacent H3 phosphorylation Oct 13, 2023 approved BIGWIG BIGWIG BIGWIG BIGWIG BIGWIG BIGWIG BROADPEAK BIGWIG BED
GSM7083373 HeLa, H3T3ph, Mitotic, Rep1 Oct 13, 2023 approved None
GSM7083374 HeLa, H3T3ph, Mitotic, Rep2 Oct 13, 2023 approved None
GSM7083375 HeLa, H3T3ph, Mitotic, Rep3 Oct 13, 2023 approved None
GSM7083376 HeLa, Input for H3T3ph, Mitotic, Rep1 Oct 13, 2023 approved None
GSM7083377 HeLa, Input for H3T3ph, Mitotic, Rep2 Oct 13, 2023 approved None
GSM7083378 HeLa, Input for H3T3ph, Mitotic, Rep3 Oct 13, 2023 approved None
GSM7083379 HeLa, H3K4me2/3, Async, Rep1 Oct 13, 2023 approved None
GSM7083380 HeLa, H3K4me2/3, Async, Rep2 Oct 13, 2023 approved None
GSM7083381 HeLa, Input for H3K4me2/3, Async, Rep1 Oct 13, 2023 approved None
GSM7083382 HeLa, Input for H3K4me2/3, Async, Rep2 Oct 13, 2023 approved None
GSM7083383 HeLa, H3K4me2/3, Mitotic, Rep1 Oct 13, 2023 approved None
GSM7083384 HeLa, H3K4me2/3, Mitotic, Rep2 Oct 13, 2023 approved None
GSM7083385 HeLa, Input for H3K4me2/3, Mitotic, Rep1 Oct 13, 2023 approved None
GSM7083386 HeLa, Input for H3K4me2/3, Mitotic, Rep2 Oct 13, 2023 approved None
GSM7083387 HeLa, CIDOP GST-TAF3-PHD WT, Async, Rep1 Oct 13, 2023 approved None
GSM7083388 HeLa, CIDOP GST-TAF3-PHD WT, Async, Rep2 Oct 13, 2023 approved None
GSM7083389 HeLa, CIDOP GST-TAF3-PHD M880A, Async, Rep1 Oct 13, 2023 approved None
GSM7083390 HeLa, CIDOP GST-TAF3-PHD M880A, Async, Rep2 Oct 13, 2023 approved None
GSM7083391 HeLa, CIDOP Input, Async, Rep1 Oct 13, 2023 approved None
GSM7083392 HeLa, CIDOP Input, Async, Rep2 Oct 13, 2023 approved None
GSM7083393 HeLa, CIDOP GST-TAF3-PHD WT, Mitotic, Rep1 Oct 13, 2023 approved None
GSM7083394 HeLa, CIDOP GST-TAF3-PHD WT, Mitotic, Rep2 Oct 13, 2023 approved None
GSM7083395 HeLa, CIDOP GST-TAF3-PHD WT, Mitotic, Rep3 Oct 13, 2023 approved None
GSM7083396 HeLa, CIDOP GST-TAF3-PHD M880A, Mitotic, Rep1 Oct 13, 2023 approved None
GSM7083397 HeLa, CIDOP GST-TAF3-PHD M880A, Mitotic, Rep2 Oct 13, 2023 approved None
GSM7083398 HeLa, CIDOP GST-TAF3-PHD M880A, Mitotic, Rep3 Oct 13, 2023 approved None
GSM7083399 HeLa, CIDOP Input, Mitotic, Rep1 Oct 13, 2023 approved None
GSM7083400 HeLa, CIDOP Input, Mitotic, Rep2 Oct 13, 2023 approved None
GSM7083401 HeLa, CIDOP Input, Mitotic, Rep3 Oct 13, 2023 approved None

Genome browser session
(e.g. UCSC)

The GEO files can be conveniently loaded into and viewed using the IGV web app at https://igv.org/app/

Methodology

Replicates

For ChIP-seq and CIDOP-seq, n=2 when experiment was essentially confirming prior results, and otherwise n=3. These were independent experiments, each starting with a different batch of HeLa S3 cells.

Sequencing depth

All sequencing was 75 bp length, single-end.

GSM7083373 HeLa, H3T3ph, Mitotic, Rep1
52900899 reads; of these:
52900899 (100.00%) were unpaired; of these:
1897451 (3.59%) aligned 0 times
28223732 (53.35%) aligned exactly 1 time
22779716 (43.06%) aligned >1 times

GSM7083374 HeLa, H3T3ph, Mitotic, Rep2
95530584 reads; of these:
95530584 (100.00%) were unpaired; of these:
1995228 (2.09%) aligned 0 times
56214798 (58.84%) aligned exactly 1 time
37320558 (39.07%) aligned >1 times

GSM7083375 HeLa, H3T3ph, Mitotic, Rep3
69747939 reads; of these:
69747939 (100.00%) were unpaired; of these:
1485796 (2.13%) aligned 0 times
39902802 (57.21%) aligned exactly 1 time
28359341 (40.66%) aligned >1 times

GSM7083376 HeLa, Input for H3T3ph, Mitotic, Rep1
73828493 reads; of these:
73828493 (100.00%) were unpaired; of these:
1060311 (1.44%) aligned 0 times
54249960 (73.48%) aligned exactly 1 time
18518222 (25.08%) aligned >1 times

GSM7083377 HeLa, Input for H3T3ph, Mitotic, Rep2
83085750 reads; of these:
83085750 (100.00%) were unpaired; of these:
834286 (1.00%) aligned 0 times
61789049 (74.37%) aligned exactly 1 time
20462415 (24.63%) aligned >1 times

GSM7083378 HeLa, Input for H3T3ph, Mitotic, Rep3
85624953 reads; of these:
85624953 (100.00%) were unpaired; of these:
867154 (1.01%) aligned 0 times
63663291 (74.35%) aligned exactly 1 time
21094508 (24.64%) aligned >1 times

GSM7083379 HeLa, H3K4me2/3, Async, Rep1
57665355 reads; of these:
57665355 (100.00%) were unpaired; of these:
643490 (1.12%) aligned 0 times
42785776 (74.20%) aligned exactly 1 time
14236089 (24.69%) aligned >1 times

GSM7083380 HeLa, H3K4me2/3, Async, Rep2
82486823 reads; of these:
82486823 (100.00%) were unpaired; of these:
833989 (1.01%) aligned 0 times
65597818 (79.53%) aligned exactly 1 time
16055016 (19.46%) aligned >1 times

GSM7083381 HeLa, Input for H3K4me2/3, Async, Rep1
55080236 reads; of these:
55080236 (100.00%) were unpaired; of these:
546552 (0.99%) aligned 0 times
41602556 (75.53%) aligned exactly 1 time
12931128 (23.48%) aligned >1 times

GSM7083382 HeLa, Input for H3K4me2/3, Async, Rep2
69767825 reads; of these:

69767825 (100.00%) were unpaired; of these:
635694 (0.91%) aligned 0 times
53021932 (76.00%) aligned exactly 1 time
16110199 (23.09%) aligned >1 times

GSM7083383 HeLa, H3K4me2/3, Mitotic, Rep1
86467416 reads; of these:
86467416 (100.00%) were unpaired; of these:
762738 (0.88%) aligned 0 times
64142534 (74.18%) aligned exactly 1 time
21562144 (24.94%) aligned >1 times

GSM7083384 HeLa, H3K4me2/3, Mitotic, Rep2
67920828 reads; of these:
67920828 (100.00%) were unpaired; of these:
761123 (1.12%) aligned 0 times
55036942 (81.03%) aligned exactly 1 time
12122763 (17.85%) aligned >1 times

GSM7083385 HeLa, Input for H3K4me2/3, Mitotic, Rep1
83257103 reads; of these:
83257103 (100.00%) were unpaired; of these:
798545 (0.96%) aligned 0 times
70941003 (85.21%) aligned exactly 1 time
11517555 (13.83%) aligned >1 times

GSM7083386 HeLa, Input for H3K4me2/3, Mitotic, Rep2
86985271 reads; of these:
86985271 (100.00%) were unpaired; of these:
938924 (1.08%) aligned 0 times
64658163 (74.33%) aligned exactly 1 time
21388184 (24.59%) aligned >1 times

GSM7083387 HeLa, CIDOP GST-TAF3-PHD WT, Async, Rep1
WT_S2_R1_001
75571644 reads; of these:
75571644 (100.00%) were unpaired; of these:
865356 (1.15%) aligned 0 times
58994701 (78.06%) aligned exactly 1 time
15711587 (20.79%) aligned >1 times

GSM7083388 HeLa, CIDOP GST-TAF3-PHD WT, Async, Rep2
52676241 reads; of these:
52676241 (100.00%) were unpaired; of these:
565053 (1.07%) aligned 0 times
40012968 (75.96%) aligned exactly 1 time
12098220 (22.97%) aligned >1 times

GSM7083389 HeLa, CIDOP GST-TAF3-PHD M880A, Async, Rep1
67504926 reads; of these:
67504926 (100.00%) were unpaired; of these:
760368 (1.13%) aligned 0 times
51765297 (76.68%) aligned exactly 1 time
14979261 (22.19%) aligned >1 times

GSM7083390 HeLa, CIDOP GST-TAF3-PHD M880A, Async, Rep2
60950098 reads; of these:
60950098 (100.00%) were unpaired; of these:
698120 (1.15%) aligned 0 times
45787946 (75.12%) aligned exactly 1 time
14464032 (23.73%) aligned >1 times

GSM7083391 HeLa, CIDOP Input, Async, Rep1
66671091 reads; of these:
66671091 (100.00%) were unpaired; of these:
618500 (0.93%) aligned 0 times
51840772 (77.76%) aligned exactly 1 time
14211819 (21.32%) aligned >1 times

GSM7083392 HeLa, CIDOP Input, Async, Rep2
67961986 reads; of these:
67961986 (100.00%) were unpaired; of these:
686113 (1.01%) aligned 0 times
51783529 (76.19%) aligned exactly 1 time
15492344 (22.80%) aligned >1 times

GSM7083393 HeLa, CIDOP GST-TAF3-PHD WT, Mitotic, Rep1
72810009 (100.00%) were unpaired; of these:
861205 (1.18%) aligned 0 times
55112849 (75.69%) aligned exactly 1 time
16835955 (23.12%) aligned >1 times

GSM7083394 HeLa, CIDOP GST-TAF3-PHD WT, Mitotic, Rep2
64029782 reads; of these:
64029782 (100.00%) were unpaired; of these:
683853 (1.07%) aligned 0 times
48625597 (75.94%) aligned exactly 1 time
14720332 (22.99%) aligned >1 times

GSM7083395 HeLa, CIDOP GST-TAF3-PHD WT, Mitotic, Rep3
50071653 reads; of these:
50071653 (100.00%) were unpaired; of these:
571250 (1.14%) aligned 0 times
37909590 (75.71%) aligned exactly 1 time
11590813 (23.15%) aligned >1 times

GSM7083396 HeLa, CIDOP GST-TAF3-PHD M880A, Mitotic, Rep1
57077748 reads; of these:
57077748 (100.00%) were unpaired; of these:
769129 (1.35%) aligned 0 times
42493257 (74.45%) aligned exactly 1 time
13815362 (24.20%) aligned >1 times

GSM7083397 HeLa, CIDOP GST-TAF3-PHD M880A, Mitotic, Rep2
51488654 reads; of these:
51488654 (100.00%) were unpaired; of these:
617406 (1.20%) aligned 0 times
38550140 (74.87%) aligned exactly 1 time
12321108 (23.93%) aligned >1 times

GSM7083398 HeLa, CIDOP GST-TAF3-PHD M880A, Mitotic, Rep3
85023554 reads; of these:
85023554 (100.00%) were unpaired; of these:
969089 (1.14%) aligned 0 times
63765867 (75.00%) aligned exactly 1 time
20288598 (23.86%) aligned >1 times

GSM7083399 HeLa, CIDOP Input, Mitotic, Rep1
58065453 reads; of these:
58065453 (100.00%) were unpaired; of these:
689189 (1.19%) aligned 0 times
43325397 (74.61%) aligned exactly 1 time
14050867 (24.20%) aligned >1 times

GSM7083400 HeLa, CIDOP Input, Mitotic, Rep2
65853660 reads; of these:
65853660 (100.00%) were unpaired; of these:
739509 (1.12%) aligned 0 times
49352215 (74.94%) aligned exactly 1 time
15761936 (23.93%) aligned >1 times

GSM7083401 HeLa, CIDOP Input, Mitotic, Rep3
71746545 reads; of these:
71746545 (100.00%) were unpaired; of these:
798614 (1.11%) aligned 0 times
53826076 (75.02%) aligned exactly 1 time
17121855 (23.86%) aligned >1 times

Antibodies

Rabbit affinity-purified polyclonal H3T3ph (B8634; Jonathan Higgins)
Rabbit monoclonal H3K4me3 (C42D8; Cell Signaling Technology #9751, Lot 10)

Peak calling parameters

H3K4me2/3 peak calling was carried out using MACS2 v2.1.1.20160309 with the --broad option and standard parameters. Peaks called in more than one replicate were retained using bedtools v2.29.2 intersect, sort and merge tools plus bedops v2.4.39110 using option --everything.

Data quality

FastQC v0.11.7 (http://www.bioinformatics.babraham.ac.uk/projects/fastqc/) and MultiQC v1.7216 were used to assess the quality of sequencing reads. All PHRED scores were > 20.

Software

For sequencing data analysis, we used FastQC v0.11.7, MultiQC v1.7216, Bowtie2 v2.3.4.2, Samtools v1.9, bedtools v2.29.2, Sambamba v0.7.1, deepTools2 v3.5.1, MACS2 v2.1.1.20160309, bedops v2.4.39, KaryoploteR v1.22.0, R v4.2.1 or v4.2.2, IGV v2.4.16, ChromHMM v1.20, and WebGestalt 2019, as detailed in the Methods.
